# The Effects of Citalopram and Thalamic Dopamine D_2/3_ Receptor Availability on Decision-Making and Loss Aversion in Alcohol Dependence

**DOI:** 10.1155/2022/5663274

**Published:** 2022-09-20

**Authors:** Todd Zorick, Kyoji Okita, K. Brooke Renard, Mark A. Mandelkern, Arthur L. Brody, Edythe D. London

**Affiliations:** ^1^Department of Psychiatry and Biobehavioral Sciences, UCLA, USA; ^2^Lundquist Institute at Harbor-UCLA Medical Center, USA; ^3^Integrative Brain Imaging Center, Japanese National Center of Neurology and Psychiatry, Japan; ^4^California University of Science and Medicine, USA; ^5^Dept. of Physics, University of California, Irvine, USA; ^6^Dept. of Nuclear Medicine, West Los Angeles VA Medical Center, USA; ^7^Dept. of Psychiatry, UCSD/VA San Diego, USA; ^8^Dept. of Molecular and Medical Pharmacology, UCLA, USA

## Abstract

Selective serotonin reuptake inhibitors (SSRIs) are commonly prescribed for patients who misuse alcohol, especially in the context of comorbid depressive symptoms. Deficits in impulse control and decision-making are linked to routine alcohol consumption and alcohol dependence. The goal of this study was to determine the effects of a single dose of citalopram on measures of impulsivity, decision-making, and/or brain dopamine receptor availability in alcohol-dependent individuals. A double-blind, placebo-controlled, within-subject, outpatient study was conducted with active alcohol-dependent (DSM-IV-TR criteria) participants (*n* = 12) and matched healthy controls (*n* = 13). Serial doses of both citalopram (40 mg) and saline were administered intravenously before laboratory tests of decision-making (Balloon Analogue Risk Task, delay discounting task, and Loss Aversion Gambling Task) and positron emission tomography with [^18^F]-fallypride to measure dopamine D_2/3_ receptor availability, separated by at least one week. Alcohol-dependent participants demonstrated greater loss aversion than healthy controls, but there were no group differences in risk taking on the Balloon Analogue Risk Task. Citalopram increased delay discounting across groups, with no group difference in the effect. There were no effects of citalopram on risk taking on the Balloon Analogue Risk Task. PET showed a negative correlation between thalamic dopamine D_2/3_ receptor availability and loss aversion across groups. The effect of citalopram to decrease the valuation of monetary reward as a function of delay raises the possibility that SSRIs can influence risky decision-making in clinical populations. In addition, these results suggest that altered thalamic dopamine signaling may play an important role in disproportionately valuing losses in patients with Alcohol Use Disorder. This trial is registered under ClinicalTrials.gov registration NCT01657760.

## 1. Introduction

Alcohol misuse represents a highly prevalent, global public health problem. In 2012, about 3.3 million deaths, or 5.9% of all global deaths, were attributable to alcohol consumption [1]. In the United States, the lifetime prevalence of Alcohol Use Disorder, as defined by DSM-5, was estimated to be 29.1 percent during 2013/2014 [2], and alcohol was the third leading preventable cause of death [3]. Substantial evidence has supported a link between faulty decision-making and addictions [4, 5]. Moreover, behavioral and neural aspects of alcoholism have been linked to impulsive decision-making and risky behavior in alcohol-dependent individuals and how these patterns differ at different stages of alcoholism dependence and recovery [6]. This study therefore focused on risky decision-making, which may affect the course of Alcohol Use Disorder and recovery. For the study presented here, the behavioral measures used were the Balloon Analogue Risk Task (BART), a delay discounting task (DDT), and the Loss Aversion Gambling Task (LAGT).

The BART is a computerized, naturalistic test in which participants make a series of choices between taking a specified amount of money or pumping a virtual balloon in order increase potential reward, each time risking explosion of the balloon and loss of winnings on that trial. Alcohol-dependent research participants take more risk than controls on the BART in some studies [7, 8], and increased alcohol consumption is associated with both priming (small doses of alcohol sufficient to elicit craving) and risky behavior on the BART [9]. In healthy control subjects, striatal BP_ND_ modulates prefrontal cortex activation while the participant is deciding to take risk and influences the number of pumps in the following trial [10].

Delay discounting tasks measure a person's devaluation of a reward as a function of delay in its receipt. Discounting represents impatience that is considered to be a form of impulsive decision-making. Participants make a series of choices for either immediate, smaller rewards or delayed, larger rewards. Higher rates of discounting are consistently exhibited by individuals with Alcohol Use Disorder [11–13] and other substance use disorders [14–16].

The phenomenon of loss aversion, that individuals subjectively value potential monetary losses more than monetary gains of equal objective value, can be measured using a paradigm developed four decades ago [17, 18]. Decreased loss aversion as compared with control groups has been observed in studies of alcohol-dependent participants in extended abstinence [19, 20]. Whereas monetary decision-making and impulsivity has been studied previously in active drinkers (e.g., [21, 22]), loss aversion via the LAGT has not been studied before in individuals with active alcohol dependence; we therefore include the LAGT in this study. The task involves probabilistic gambling whereby the participant decides whether to accept individual bets winning (or losing) amounts from $5 to $50, which would be decided via a coinflip.

Individuals with Alcohol Use Disorder commonly suffer from psychiatric comorbidities, which contribute to complexity in the manifestation of and treatments for their addiction [23]. The frequent comorbidity of depressive symptoms with Alcohol Use Disorder provide a foundation for evaluating the use of selective serotonin reuptake inhibitors (SSRIs) in patients with this disorder. Notably, low cerebrospinal fluid serotonin levels are linked to greater alcohol intake [23–25]. However, SSRI treatment has not consistently reduced alcohol use by individuals who misuse it [23, 26, 27]. Most relevant studies have shown no reduction in alcohol consumption or craving with SSRIs [27–29], and citalopram treatment was associated with worse outcomes for some measures of alcohol dependence in one study [30]. Given that SSRIs may be associated with increased aggression and suicidality in certain populations [31, 32], it is reasonable to question the risk/benefit profile of SSRIs in Alcohol Use Disorder.

Among SSRIs, citalopram has high specificity and selectivity for serotonin uptake [33]; however, several studies using PET have demonstrated modulation of intrasynaptic striatal dopamine concentration by administration of SSRIs, likely via an indirect mechanism [34, 35]. Most relevant to the present study is the observation that a single intravenous dose of citalopram produced a modest decrease in striatal dopamine D_2/3_ receptor BP_ND_, consistent with an increase in intrasynaptic dopamine concentration [36]. Although the neural circuitry responsible for an SSRI increasing impulsivity is unclear, a rodent study utilizing the antidepressant milnacipran showed that high doses were associated with increased behavioral aggression and impulsivity along with increased dopamine release in the nucleus accumbens, which the authors speculated could be due to enhanced serotonergic transmission in the nucleus accumbens shell [37].

The study presented here utilized a within-subject, double-blind, crossover design in which the effects of intravenous (iv) citalopram (40 mg) were compared to those of saline placebo on decision-making and D_2/3_ receptor BP_ND_ in research participants who met criteria for alcohol dependence (DSM-IV-TR) and comparable healthy control participants. Decision-making was tested in using the BART, DDT, and LAGT. Performance on the BART and a DDT has shown associations with dopamine D_2/3_ receptor availability (nondisplaceable binding potential, BP_ND_) on positron emission tomography (PET) scanning [10, 14]. We therefore also measured dopamine D_2/3_ receptor BP_ND_ with PET. The goals were to evaluate effects of citalopram on reward-based decision-making tasks and to determine whether potential findings would involve effects on D_2/3_ receptor BP_ND_. We anticipated that alcohol-dependent research subjects (compared to control subjects) would exhibit decreased striatal D_2/3_ receptor BP_ND_ and that striatal D_2/3_ receptor BP_ND_ would correlate with impulsive decision-making on the BART, DDT, and LAGT.

## 2. Method

### 2.1. Overview

The Veterans Administration Greater Los Angeles IRB approved all procedures. As described in a previous study by our group [38], recruitment was accomplished by internet ads and recruitment fliers. The study enrolled participants with active alcohol dependence (DSM-IV-TR criteria; AD group) and healthy control participants (HC group) who were demographically comparable. Study procedures included a screening day, structural MRI scan, and two test days that included test compound administration and PET scanning. The test compound was either citalopram (40 mg iv) or saline placebo (counterbalanced order, with at least one week between procedure days). After enrollment, participants could continue in the study only if they had exhaled breath alcohol concentration of zero (confirmed by a breathalyzer) and low alcohol withdrawal scores on all subsequent procedure days. This study is novel because it utilized active alcohol-dependent individuals without a history of severe alcohol withdrawal symptoms, who were not seeking treatment, but were able to participate in study procedures while maintaining temporary abstinence for at least 16 hours per procedure day.

On infusion/PET scan days, procedures were carried out in the following order: (1) breathalyzer testing, the Clinical Institute Withdrawal Assessment for Alcohol (CIWA) scale; (2) intravenous citalopram or saline placebo infusion (over 1 hour); (3) DDT, BART, and LAGT (in the stated order; 45 min of testing starting 30-60 min after citalopram/saline infusion); (4) [^18^F]-fallypride PET scanning (3 hr); (5) assessment for adverse events before sending participants home for the day.

### 2.2. Participants

The participant target groups consisted of AD and HC, screened with the SCID-IV to exclude participants with Axis I psychiatric diagnoses within the previous 6 months. Review of VA electronic health records and current SCID-IV testing excluded potential participants with any recent mental illness, including depression [38]. Exceptions included nicotine dependence in both groups and alcohol dependence in the AD group. Qualifying participants were 21-55 years of age and had not used any psychoactive medications in the 30 days before enrollment. All participants had normal physical exams, EKGs, and laboratory studies in screening. T.Z., a Board-Certified psychiatrist, interviewed and reviewed all participants as part of screening.

Twenty AD and 20 HC participants were screened for participation in the study. We enrolled 14 AD and 17 HC participants. Of these, 12 AD participants and 13 HC participants completed the study. Due to PET scanner malfunction or radiosynthesis failure, eight AD participants (seven completed both scans, one had one good scan) and seven HC participants (six completed both scans, one had one good scan) had useful PET scan data. All participants had negative alcohol breathalyzer results and saliva toxicology (testing for opiates, benzodiazepines, amphetamine, cocaine, and cannabinoids) at screening. Participants were compensated up to $30 per session for BART and LAGT performance. No compensation was provided for DDT performance because an a prior report showed no difference in performance on the DDT whether real or hypothetical money was provided [39].

### 2.3. Assessments

The Beck Depression Inventory-II was employed to measure current depressive symptoms (Beck and Steer, 1996). CIWA [40] scores < 10 were required to ensure safe participation. The decision-making tasks were conducted under standard conditions in the following order: DDT, BART, and LAGT.

The BART involves earning a small reward (typically $0.05) per pump of a balloon until a random threshold where the balloon bursts. If the balloon breaks, the participant will receive no money for that balloon. If the participant chooses to “cash-out” before breaking the balloon, they earn an allotted amount of money for that trial. The BART was carried out using the PEBL BART implementation, utilizing the same procedures as the original study [41]. More detail concerning the BART procedure is described elsewhere [10, 42]. We utilized the measure of mean clicks per balloon that did not explode (“adjusted pumps”) as our primary outcome measure [42].

The DDT was carried out using pen and paper and the Monetary Choice Questionnaire [43]. Mazur's *k* values were estimated for each participant, with larger *k* values corresponding to larger discounting of future rewards (greater temporal discounting). Participant-specific *k* values were calculated as described for each questionnaire [43], with the natural logarithmic transform used to improve parametric statistical analysis (e.g., [44]).

Lastly, on the LAGT, we utilized two 60 bet blocks per session to estimate loss aversion coefficients. For example, subjects considered a 50/50 bet for the chance to win $10 or lose $5. The LAGT was conducted using MATLAB Psychtoolbox implementation [45], with linear regression of choice data to generate loss aversion coefficients following [46].

### 2.4. MRI and PET Analysis

The specifics of structural MRI scans obtained on a 3-T Siemens tomograph and [^18^F]-fallypride (~5 mCi) PET scans have been described in depth in prior research [38, 47]. [^18^F]-Fallypride was synthesized in the VA Los Angeles radiochemistry production facility, as reported in prior research [48]. Investigational New Drug (IND) approval (number 78,226) for the use of iv citalopram was obtained from the FDA. Quality control, completed for each radiotracer batch, demonstrated apyrogenicity, ≥98% radiochemical purity, and specific activity > 1 Ci/*μ*mol at injection. Using nonspecific fallypride binding in the cerebellum (known to have minimal dopamine receptor expression) as a reference tissue, we estimated tissue-specific dopamine D_2/3_ receptor density via the two-tissue compartment model by calculating the ratio of specific to nonspecifically bound radiotracer binding potential (dopamine D_2/3_ receptor BP_ND_) after Innis et al. [49].

### 2.5. PET Volume of Interest Analysis

Standardized subcortical volumes of interest (VOIs) were automatically produced from structural MRI using FSL FIRST. VOIs were determined for the striatum (including the caudate nucleus, putamen, and nucleus accumbens as separate VOIs), globus pallidus, and thalamus. Binding potential (BP_ND_) for each VOI was calculated via a simplified reference tissue modeling, utilizing the cerebellum as the reference region (PMOD Technologies Ltd., Zurich, Switzerland) (see [38, 47, 50] for more information).

### 2.6. Statistics

All statistical analyses were done using R [51]. Baseline statistical tests (e.g., ANOVA) were done using the R Stats in the base package. Mixed linear modeling with compound symmetry matrices were used to analyze participant data collected within subjects, in order to better model missing data, using the “nmle” package [52]. Associations between variables were assessed via Pearson's product-moment correlations.

## 3. Results

### 3.1. Demographics

The demographics and clinically relevant information from the participants on study intake are presented in Table 1. The AD and HC groups did not differ in sex/gender, ethnic compositions, age, years of education, or intake CIWA score (Table 1). AD participants consumed 6.0 ± 3.3 (mean ± s.d.) drinks per day over the 90 days prior to entering the study, whereas HC participants consumed 0.1 ± 0.2 drinks per day (*F*_(1, 23)_ = 36.7, *p* < 0.001; Table 1). Seven of the 12 AD participants smoked cigarettes, but none of the HC participants were current smokers (*F*_(1, 23)_ = 21, *p* < 0.001; Table 1).

### 3.2. Adverse Events

Adverse events were compared between citalopram and saline infusion days, across participant groups (Table 2). While adverse events were relatively common, with at least half of participants complaining of dizziness, fatigue, and/or nausea during the citalopram infusions, about 20% of participants complained of at least one of these symptoms during the saline infusions. However, only the subjective experience of nausea was present significantly more frequently in citalopram compared to saline conditions (8 vs. 1 out of 10 participants, *p* < 0.05; Table 2). All adverse events were mild, required no treatment, and spontaneously resolved in less than 24 hours; there were no serious adverse events during the study.

### 3.3. BART

All 12 AD participants and 12 HC participants completed the BART on both study arms, whereas one HC participant missed the BART on one of the study arms. AD participants had an average of 11.5 ± 6.9 pumps per balloon, and HC participants averaged 12.4 ± 6.3 pumps; by condition, participants receiving iv citalopram averaged 12.3 ± 6.9 pumps, while participants receiving saline placebo averaged 11.7 ± 6.3 pumps. There was no difference via linear mixed effects modeling on BART scores between AD and HC participants (*t* (23) = −0.33, *p* = 0.75) nor between participants receiving either citalopram or saline (*t* (23) = −1.4, *p* = 0.17), and there was no group × condition interaction (*t*(21) = 1.45, *p* = 0.16).

### 3.4. DDT

**Figure 1:**
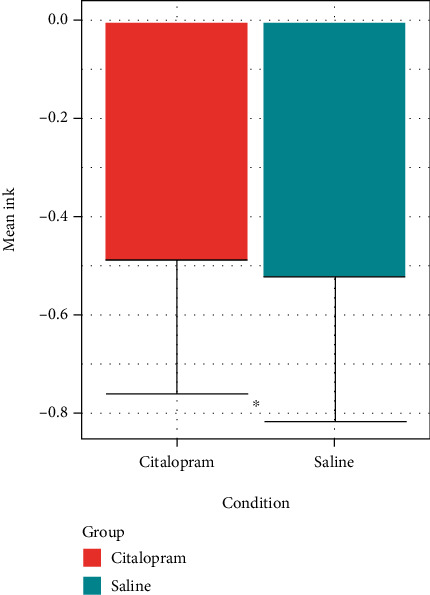
Mazur's *k* values for discount delay task, by condition. Bars represent mean (s.d.) natural logarithmically transformed *k* values calculated from participants in each condition. Participants displayed a greater degree of temporal discounting (larger *k* value) in the citalopram compared to saline conditions. Citalopram-citalopram (40 mg) iv infusion; saline-matched saline placebo. ^∗^ indicates *p* < 0.05 for effect of condition by linear mixed effects modeling.

All 13 HC participants and 11 of the AD participants completed DDT questionnaires on both study arms, whereas one AD participant completed a questionnaire on only one of the arms. Using the log transformed *k* values from DDT answers for participants, AD participants had a mean ln(*k*) value of −0.45 ± 0.24, whereas HC participants' mean ln(*k*) value was −0.56 ± 0.31. Across participant groups, participants in the saline condition had a mean ln(*k*) value of −0.54 ± 0.29, while participants receiving citalopram had a corresponding mean ln(*k*) of −0.49 ± 0.27 (see Figure 1). Using linear mixed effects modeling, there was no effect of group (AD vs. HC; *t* (22) = −0.86, *p* = 0.4), but there was an effect of condition (citalopram vs. placebo; *t*(21) = −2.2, *p* = 0.04; Figure 1), with no group × condition interaction (*t*(21) = 0.82, *p* = 0.42). This effect of condition was confirmed with nonlog transformed *k* values, showing that participants receiving citalopram had a raw mean *k* value of 0.634 ± 0.158, while in the saline condition, their mean *k* value was 0.616 ± 0.168 (*t*(22) = −2.11, *p* = 0.046). Thus, across groups, participants receiving citalopram tended to have larger *k* values in the DDT, indicating a greater tendency to discount future rewards, with no difference between AD and HC subjects generally. However, the tendency of AD participants to have a trend to lower ln(*k*) values compared to the HC participants in this study is consistent with the direction of the effect in larger studies.

### 3.5. LAGT, Alcohol Dependence, and IV Citalopram

**Figure 2:**
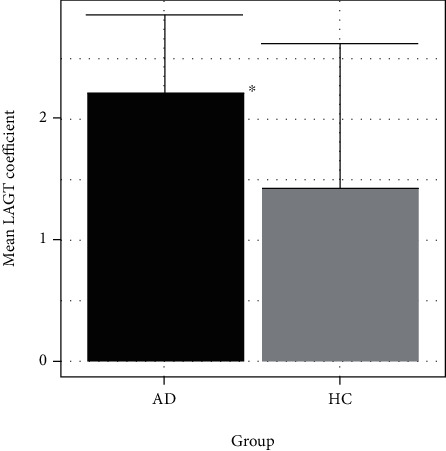
LAGT coefficient values, by participant group. Bars represent mean (s.d.) LAGT coefficients from each participant group. AD: alcohol-dependent participants; HC: control participants. ^∗^ indicates *p* < 0.05 for effect of group by linear mixed effects modeling.

It was possible to estimate LAGT coefficients for both study arms for 11 of the AD participants and 10 of the HC participants; the other participants completed the LAGT task, but their answers were so inconsistent as to preclude accurate estimation of LAGT coefficients. Mean loss aversion coefficients (*λ*) for the HC group were 1.4 ± 1.2, whereas for the AD participants, they were 2.2 ± 0.64 (Figure 2). Across both participant groups, the mean *λ* value was 1.9 ± 1.3 for the citalopram condition and 1.7 ± 0.8 for the saline placebo condition. Linear mixed effects modeling showed a main effect of group (AD vs. HC; *t*(19) = −2.67, *p* = 0.015; Figure 2), but no effect of condition (citalopram vs. placebo; *t*(19) = −1.55, *p* = 0.14), and no group × condition interaction (*t*(19) = 1.4, *p* = 0.18). Given the trend to increased BDI in AD participants on screening, we also controlled for infusion day BDI scores and found that the observed difference in *λ* between AD and HC groups remained significant (*t*(14) = −2.7, *p* = 0.017). Therefore, the mean *λ* value for the AD group was larger than the mean *λ* value for the HC group, indicating that the AD participants had a greater degree of loss aversion, with no effect of citalopram infusion.

### 3.6. Brain Region-Specific Dopamine D_2/3_ Receptor BP_ND_

There were no differences between groups, conditions (citalopram vs. placebo), or group × condition interactions for mean dopamine D_2/3_ receptor BP_ND_ for caudate, nucleus accumbens, or putamen via linear mixed effects modeling (Table 3). There was a trend to a group difference in the globus pallidus (with no effect of condition or group × condition interaction; 8.8 ± 2.5 for AD, 11.3 ± 3.5 for HC; *t*(13) = 2.75), but this difference did not survive Bonferroni correction for multiple comparisons (*p* = 0.08 after correction). After Bonferroni correction, there was a significant group difference in thalamic dopamine D_2/3_ receptor BP_ND_ (2.0 ± 0.48 AD, 2.7 ± 0.49 HC, *t*(13) = 3.1, adjusted *p* = 0.044), with no effect of condition, and no group × condition interaction. Therefore, the tendency is for thalamic and globus pallidus dopamine D_2/3_ receptor BP_ND_ to be lower in AD than in HC. Given the group difference for AD and HC in thalamic dopamine D_2/3_ receptor BP_ND_, and the trend for globus pallidus dopamine D_2/3_ receptor BP_ND_, we utilized dopamine D_2/3_ receptor BP_ND_ values for these two regions in subsequent correlation analyses.

### 3.7. Correlation between Loss Aversion Coefficients and Thalamic and Globus Pallidus BP_ND_

**Figure 3:**
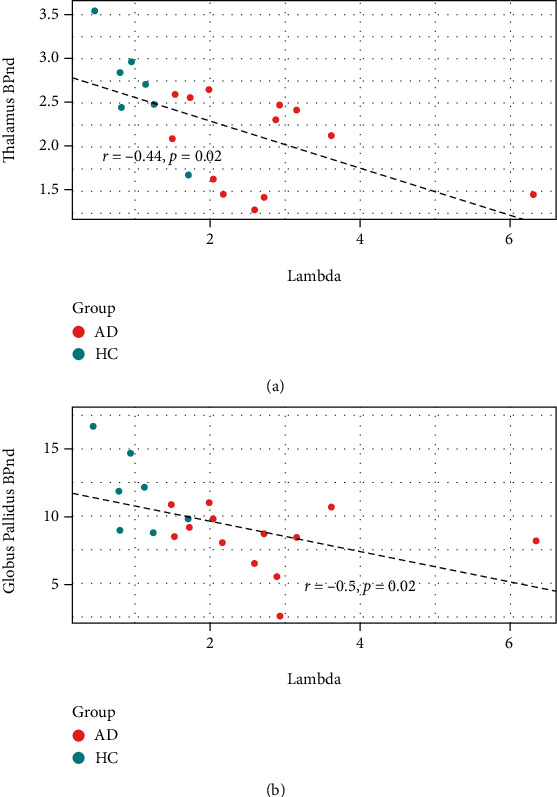
Correlation plots of LAGT coefficients (lambda) vs. regional [^18^F]-fallypride binding potential, by group. Each data point represents a participant in a particular study arm. (a) Thalamic BP_ND_ vs. lambda values for participants. (b) Globus pallidus BP_ND_ vs. lambda values for participants. Inscribed values represent Pearson's product-moment correlation values, along with the corresponding *p* values. Group: AD: alcohol-dependent participants (orange circles); HC: control participants (blue circles). Correlations were robust to removing the data for subject with the highest lambda value (>6; *p* = 0.015 for thalamus, *p* = 0.0031 for globus pallidus).

Since both loss aversion coefficients and thalamus (and globus pallidus) dopamine D_2/3_ receptor BP_ND_ values differed between AD and HC participants, we tested whether these values were correlated among participants (Figure 3). Across participants, both thalamic dopamine D_2/3_ receptor BP_ND_ (Figure 3(a); *r* = −0.44, *p* = 0.02) and globus pallidus dopamine D_2/3_ receptor BP_ND_ (Figure 3(b); *r* = −0.5, *p* = 0.02) were correlated negatively with loss aversion coefficients (*λ* values). These results were robust to removing the data for the subject with the extreme *λ* value (>6; *r* = −0.55, *p* = 0.014 for thalamus, *r* = −0.64, *p* = 0.0031 for globus pallidus). There were no significant correlations between ln(*k*) values and thalamus (*r* = −0.19, *p* = 0.3) or globus pallidus (*r* = −0.15, *p* = 0.4) dopamine D_2/3_ receptor BP_ND_ values. There were also no significant correlations between BART pumps per balloon and thalamus (*r* = −0.21, *p* = 0.3) or globus pallidus (*r* = −0.25, *p* = 0.21) dopamine D_2/3_ receptor BP_ND_ values.

## 4. Discussion

### 4.1. Balloon Analogue Risk Task

No significant difference was present on the BART between AD and HC participants. A prior study reported greater risk taking on the BART by alcohol-dependent individuals vs. controls in a slightly larger sample (*n* = 17 per group) [7]. A salient difference between the two studies is that the previous study included alcohol-dependent participants who were taking psychiatric medications, including “anxiolytics” (at least 9 out of 17, whereas none of their control group had any psychotropic medication use), a difference that may have impacted the results [7]. However, benzodiazepines have variable effects on risk taking in healthy volunteers (cf. [53] with no effect on BART, other measures of risk taking affected in [54]), whereas a study in patients with bipolar illness (where anxiolytic use is common) showed abnormalities in BART behavior after popped balloons [55].

Intravenous citalopram, compared to saline, did not affect either BART performance or striatal dopamine D_2/3_ receptor BP_ND_ in our sample. Prior research has demonstrated via PET analysis that striatal dopamine D_2/3_ receptor BP_ND_ correlates with modulation of prefrontal cortex activation by level of risk and reward during the decision to make risky pumps and with modulation of ventral striatal activation during the decision to “cash-out” on the BART [10]. D_2/3_ receptor BP_ND_ in the striatum is also negatively associated with number of pumps in the following trial and modulation of prefrontal cortex activation during risky behavior [10].

### 4.2. Delay Discounting Task

The DDT revealed no difference in performance between AD and HC groups in this study. Alcohol dependence has been consistently linked with greater delay discounting across cross-sectional and longitudinal studies [11, 12]. A meta-analysis of delayed discounting in studies of substance use disorder (and Alcohol Use Disorder specifically) showed that there was likely to be a *Cohen'sd* effect size of ~0.5 for “clinical” populations with alcohol dependence compared to controls, with considerable heterogeneity in individual trials [15]. Therefore, the current study likely was underpowered to detect any difference in DDT between study groups, given the current sample size (power ~ 22% given *d* = 0.5, and *p* = 0.05; [56, 57]).

In prior work, delay discounting was significantly negatively correlated with striatal dopamine D_2/3_ receptor BP_ND_ in a combined sample of 27 methamphetamine-dependent individuals and 27 HC participants and among the methamphetamine users alone [14]. However, no correlation was found between DDT and regional dopamine D_2/3_ receptor BP_ND_ in this study, possibly reflecting lack of adequate power due to small sample size. Nonetheless, citalopram increased delay discounting across subject groups in this study. This effect is noteworthy because higher rates of discounting are indicative of greater impulsivity in Alcohol Use Disorder [12]. If citalopram and/or other SSRIs are shown to reliably increase delay discounting in future studies, this phenomenon may explain the difficulty experienced by some substance users in reducing substance use while taking SSRIs [58].

### 4.3. Loss Aversion Gambling Task

Greater loss aversion as measured by the LAGT was observed in the AD group, which is a novel finding. A potential explanation for this result may lie in the complex interplay between alcohol dependence and depression. Alcohol abuse and depressive symptoms are highly comorbid. Depressed individuals have greater loss aversion than healthy controls, involving hyperresponsiveness in the anterior insula in assessments of responses to experimental monetary loss [59, 60]. However, participants with Major Depressive Disorder or other mood disorders were excluded from this study, the groups did not differ in BDI scores at study entry, and results were robust to controlling for infusion day BDI scores. Thus, the presence of depressive symptoms does not likely explain increased loss aversion in the AD group, compared to controls. Two previous studies with participants with Alcohol Use Disorder in remission/after detoxification showed that patients with Alcohol Dependence/Use Disorder had less loss aversion compared to controls [19, 20]. It is possible that the unexpected finding here with the opposite effect may be due to active alcohol users differing in loss aversion compared to research subjects in extended abstinence, and/or it may be related to the effect of alcohol on disturbing the hypothalamic-pituitary-adrenal gland stress activation [61]. A recent study of adolescents with laboratory-induced alcohol intoxication showed that greater loss aversion was linked to an increased family history of alcohol problems [62]; therefore, genetic predisposition for alcohol misuse may relate to increased loss aversion. A review of the evidence about HPA dysregulation in early abstinence from alcohol demonstrates the complex relationship between alcohol dependence and HPA/stress regulation. While early abstinence in alcohol dependence is associated with blunted HPA axis responsiveness and generally elevated levels of cortisol, lower levels of cortisol are associated with increased craving for alcohol and tendency to relapse (reviewed in [63]). One study found that the combination of yohimbine, an alpha 2 adrenergic receptor antagonist, and hydrocortisone (both of which activate physiological stress responses) in healthy adults reduced loss aversion [64]. However, another similar study in healthy adults showed no change in loss aversion [65]. Given the clearly complex relationship between stress, HPA axis responsiveness, and early abstinence in alcohol dependence, more investigation will be needed to clarify these phenomena. In any event, our current results would need to be replicated in larger studies, to see if this phenomenon is more generally observable in active alcohol misusers.

In terms of genetic correlates of loss aversion, genetic loci found to be important for loss aversion include BDNF secretion, striatal dopamine D_2/3_ receptor expression, and serotonin transporter gene expression [66–68]. A study of behavioral traits in a large database of Swedish twins produced a heritability estimate of 23% for loss aversion, with the majority of variation unexplained by either shared genetics or environment [69].

### 4.4. Loss Aversion and Globus Pallidus/Thalamic Dopamine D_2/3_ Receptor BP_ND_

This study demonstrated negative correlation between thalamic and globus pallidus dopamine D_2/3_ receptor BP_ND_ and loss aversion coefficients, across subject groups. These findings indicate that greater loss aversion associates with decreased thalamic dopamine binding potential, as has been previously shown in alcohol dependence (cf. [38]). A previous study showed that thalamic noradrenergic receptor BP_ND_ as assessed via PET scanning negatively correlated with loss aversion [70], thus indicating that thalamic monoamine signaling is implicated in loss aversion more generally, possibly through a decrease in loss prediction signaling.

The finding of a correlation between globus pallidus dopamine D_2/3_ receptor BP_ND_ and loss aversion is novel and to our knowledge has not previously been reported. The globus pallidus has been less implicated in studies of decision-making and reward than other midbrain structures such as the thalamus, ventral striatum, and ventral pallidum [71]. Induced globus pallidus lesions in rodents have been shown to produce hyperkinetic disorders than were designed to mimic Huntington's disease, with many lesions resulting in a differential behavioral hyperkinesis in response to aversive stimuli versus rewarding stimuli (bitter versus sweet oral liquid solutions [72]). Therefore, there is some indication that the globus pallidus may be involved in differential motor responses to aversive and rewarding stimuli [72].

Correlations between loss aversion coefficients and brain activation via fMRI have been found in the bilateral ventral striatum, superior prefrontal cortex, and right inferior parietal cortex [46]. Another study that addressed individual neural differences in loss aversion found that the loss aversion network may include the amygdala, thalamus, striatum, and posterior insula [73]. A separate MRI study found that exposure to fearful faces increased the saliency of losses compared to normal faces, which was mediated by increased amygdala activity [74]. The implication of the thalamus in the loss aversion network is noteworthy given the association we report herein between high loss aversion and low thalamic dopamine D_2/3_ receptor BP_ND_. Therefore, thalamic dopamine signaling may serve as an important mediator of loss aversion.

We previously showed that cue-induced craving for alcohol was negatively correlated to thalamic dopamine D_2/3_ receptor BP_ND_ [38], in a study focused on cue-induced craving for alcohol using a subset of the same data presented here. Since high loss aversion is also associated with low thalamic dopamine D_2/3_ receptor BP_ND_, loss aversion (along with craving) may be a possible biomarker for relapse liability.

## 5. Limitations

One limitation in this study was the small sample size, which limits the power to detect group and medication differences on decision-making tasks. The small sample size was underpowered to account for any effect of clinical data of the participants such as severity of alcohol dependence, length of heavy alcohol use, previous history of depression and/or comorbid psychiatric illness, or medical comorbidities. We excluded participants with active psychiatric diagnoses, and comorbid substance use and those taking psychotropic medications, which may limit the generalizability of these findings to broader clinical populations; however, our approach is likely to have provided a participant group with more reproducible effects to experimental procedures. Given that SSRIs take weeks to show clinical efficacy, a single dose of citalopram was likely unable to produce maximal effects on measures of decision-making. While the BART purports to assess risky decision-making, it is also dependent upon the ability to learn under uncertainty [75]; improved tests of risky decision-making could assess choices under known probability distributions (e.g., [76]). As a high-affinity ligand for dopamine D_2/3_ receptors, [^18^F]-fallypride PET scanning produces an estimate of tissue-specific receptor concentration but is relatively insensitive to changes in dopamine signaling. Finally, we are unable to fully account for any effect of active smoking on tasks of decision-making and future discounting, and in this study, as has frequently been demonstrated in the literature, there was extensive collinearity between alcohol dependence and smoking [38, 77], and smoking was prevalent in our AD but not the HC sample.

## 6. Conclusion

Overall, loss aversion was higher in AD patients relative to healthy controls. PET analysis revealed a negative correlation between thalamic dopamine and loss aversion. High loss aversion may be related to alterations in dopamine receptor activity seen in Alcohol Use Disorder. The observed combination of high loss aversion and the higher risk taking related to gains present in alcohol dependence [78] may indicate dysfunctional decision-making. Individuals with active alcohol dependence may therefore be impaired in their ability to assess financial risk/reward ratios. Moreover, the findings point to a role of signaling through thalamic dopamine D_2/3_ receptors in the behavioral phenomenon of loss aversion.

Administration of iv citalopram was linked with higher delay discounting in both alcohol-dependent patients and healthy controls. Therefore, acute SSRI administration may increase impulsive responding, favoring immediate rewards, as measured by delay discounting tasks. This study demonstrates that both iv citalopram and low thalamic dopamine are correlated with altered reward-based decision-making. Future studies should examine the effect of chronic oral antidepressants on impulsivity, include delay discounting, especially in patients with Alcohol Use Disorder. If future studies also demonstrate that SSRI treatment can alter delay discounting, this would be an important treatment risk that should be communicated to patients. Thalamic dopamine abnormalities associated with alcohol dependence may have major implications on impulse control and risky behavior in this population.

## Figures and Tables

**Table 1:** Demographic and study entry data.

Category	AD	HC	Statistic^∗^	*p*
Male	8 (80%)	5 (50%)	3.7	0.35
White	5 (50%)	2 (20%)	1	1
Black	3 (30%)	4 (40%	1	1
Age	43.2 (8.1)	38.3 (8.9)	0.85	0.37
BDI	9.5 (8.5)	4.4 (4.6)	2.8	0.11
Education	12.9 (1.5)	13.8 (1.5)	1.8	0.2
EtOH drinks/day	6.0 (3.3)	0.13 (0.19)	*36.7*	*<0.001*
Current smokers	7 (70%)	0 (0%)	*0*	*0.003*
CIWA	0.5 (0.7)	0.8 (1.0)	0.6	0.46

AD: alcohol-dependent group; HC: healthy control group. Categorical information: values represent number (percent). Numerical information: values represent mean (s.d.). Statistics represent Fisher's exact test odds ratios (categorical variables: gender, ethnicity, smokers) and ANOVA *F* statistic values (continuous variables). Italics: *p* < 0.005.

**Table 2:** Adverse event table.

Group	Dizziness	Fatigue	Muscle tension	Nausea
AD	5 (50%)	7 (70%)	2 (20%)	8 (80%)
HC	2 (20%)	2 (20%)	2 (20%)	1 (10%)
OR (95% CI)	0.34 (0.03 to 2.5)	0.21 (0.02 to 1.4)	1 (0.07 to 15.2)	*0.08* ^∗^ *(0.002 to 0.75)*

AD: alcohol-dependent group; HC: healthy control group. Statistics generated using Fisher's exact test. Values for each AE represent number (percent) reporting. Italics^∗^: *p* < 0.05.

**Table 3:** Region-specific BP_ND_s between AD and HC participants.

Region-specific BPs between AD and HC participants					
Region	AD	HC	Statistics		
			Group	Condition	Group × condition
Caudate	22.1 (4.1)	23.7 (5.2)	2	0.46	-1.8
Globus pallidus	8.8 (2.5)	11.3 (3.5)	*2.75*	0.25	-2
Nucleus accumbens	19.6 (5.8)	19.1 (7.8)	0.61	-0.36	0.33
Putamen	25.7 (4.3)	28.3 (6.0)	2.2	0.48	-1.7
Thalamus	2.0 (0.48)	2.7 (0.49)	*3.1* ^∗^	-0.26	-1.9

AD: alcohol-dependent group; HC: healthy control group. Values represent *t* statistics from linear mixed effects model analyses. Italics: *p* < 0.1; ^∗^: *p* < 0.05 after Bonferroni correction for multiple comparisons.

## Data Availability

Processed data in spreadsheets will be freely available for download from github.com upon manuscript publication.
